# A case of spotted fever group rickettsiosis imported into the United Kingdom and treated with ciprofloxacin: a case report

**DOI:** 10.1186/1752-1947-2-98

**Published:** 2008-04-03

**Authors:** Rifat Rashid, Alessandro C Pasqualotto, David W Denning

**Affiliations:** 1 Wythenshawe Hospital and The University of Manchester, UK

## Abstract

**Introduction:**

Spotted fever group rickettsioses are an interesting group of infections, which are increasing in incidence worldwide.

**Case presentation:**

Here we describe an imported case to the United Kingdom occurring in a patient who had recently visited Kruger National Park in South Africa – a highly endemic area for *Rickettsia *infections. Initial treatment with doxycycline failed but the patient made a prompt recovery after commencement of ciprofloxacin.

**Conclusion:**

This finding raises the possibility that there are resistant strains of *Rickettsia *present.

## Introduction

Rickettsiae are obligate intracellular Gram-negative bacteria causing acute febrile, zoonotic diseases. Rickettsiosis is an endemic condition in many areas of the world, and ticks have an important role amongst the various arthropods acting as vectors [1]. As each tick species has a preference for particular environmental conditions, tick-borne diseases are habitually restricted to specific geographic areas. African tick bite fever, caused by *Rickettsia africae*, is probably the most commonly encountered agent of rickettsiosis in travel medicine. This condition is endemic in large parts of rural Africa [2] but is rarely seen in the United Kingdom.

Although most patients with rickettsiosis have a benign and self-limiting course, complications may occur. These include prolonged fever [3,4], reactive arthritis [3], acute neuropsychiatric symptoms [5], sub-acute neuropathy and acute myocarditis [6]. Proper therapy is considered essential for rapid recovery and prevention of complications [1]. However, there is no randomised controlled trial data to guide treatment. Doxycycline has historically been considered the drug of choice, and most infected individuals show prompt response when treated with this agent. Here we report a patient with spotted fever group rickettsiosis who failed to respond to doxycycline but who had a rapid response to treatment with ciprofloxacin.

## Case presentation

A 49-year-old immunocompetent Caucasian woman presented with a 3 week history of fever, shivers, breathlessness and weakness. These symptoms started 2 days after she returned from a trip to South Africa and Zambia, which included a visit to Kruger National Park. During this period, she had been fully compliant with anti-malarial prophylaxis (Malarone). She complained of headaches and light-headedness, which were worse in the evenings and substantially impaired her ability to concentrate.

**Figure 1:**
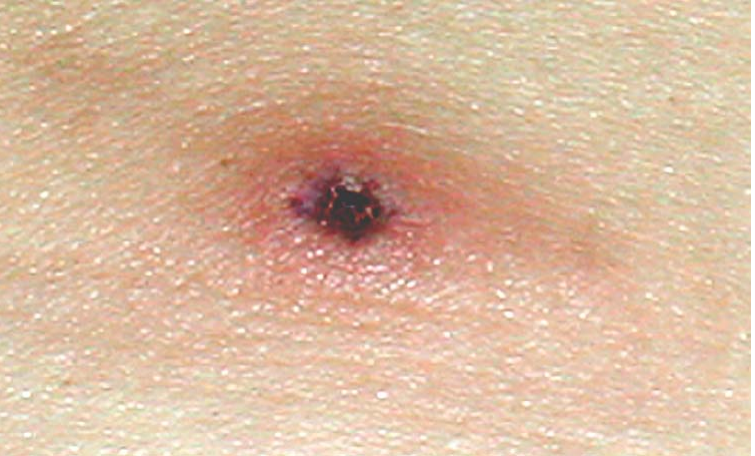
***Tache noire*** **lesion seen on the left lower abdomen 8 days after the start of the illness (colour reproduction).**

On the first day of symptoms she noticed a painful swelling in the right side of her groin, which gradually increased in size. A large lesion on her left lower abdomen was also observed, which evolved from an initial 'white head' to reveal an eschar that gradually increased in size (Figure 1). On the next 2 weeks several vesicular skin lesions emerged on her legs, right arm and abdomen. She manifested profound anorexia and discomfort in the left hypochondrium. Musculoskeletal pain and insomnia were very prominent at this stage.

When seen at our hospital, a left lower abdominal wall *tache noire *lesion was noted. In addition, there were 20 small vesicular lesions on her trunk, and she had right sided painful inguinal lymphadenopathy. Routine bloods were all normal and malarial films were negative. C-reactive protein was slightly raised at 29. Blood samples were collected for serological studies on initial presentation and 2 weeks later (Table 1). A working diagnosis of African tick bite fever was made and the patient was started on doxycycline 100 mg twice daily. On the 3^rd ^day of treatment new skin lesions on both her lower limbs were observed, fever and lassitude persisted and new visual floaters occurred. Therapy was switched to ciprofloxacin 500 mg twice daily. She responded within 24 hours and her symptoms resolved completely after 7 days of therapy. Retrospective serology performed with a commercial immunofluorescence assay confirmed recent rickettsial infection. Serological tests for individual *Rickettsia *species were not available.

**Table 1:** Patient's serology to Spotted Fever Group Rickettsioses (Porton Down specialist pathology laboratory).

	Reciprocal Titres	
	
Moment of sampling	Ig G	Ig M
	
At clinical presentation	Negative	Negative
After 10 days	≥256	Negative

## Discussion

The diagnosis of spotted fever group rickettsiosis is difficult and generally performed retrospectively. Biochemical abnormalities are usually those of acute phase reactions; mild and transient lymphopenia, thrombocytopenia and liver enzyme elevation can be observed. In addition, the offending organisms cannot be cultivated in cell-free media but can grow in yolk sacs of developing chicken embryos and in cell cultures [2], methods of little applicability in clinical practice. The diagnosis of rickettsiosis is therefore usually established using serological tests, such as immunofluorescence assays [2,7]. Since cross-reaction may occur between *R. africae *and *R. conorii*, the diagnosis of African tick bite fever using immunofluorescence can be presumptive only. Additional tests such as cross-adsorption studies or Western blotting can provide supplementary information. Many centres also increasingly perform polymerase chain reaction (PCR) tests as a very sensitive and specific tool to detect *R. conorii *and *R. africae *in a variety of clinical samples, particularly inoculation eschar biopsies [2]. However, the occurrence of an inoculation eschar with associated regional lymphadenopathy – as occurred in our patient – makes the diagnosis of African tick bite fever highly probable.

As is the case with most common rickettsioses, African tick bite fever usually manifests with acute non-specific flu-like symptoms which include fever, nausea, myalgias and headache. The time lag from tick bite to symptom onset is usually 5–7 days [2,6] but may be as long as 12 days [8]. Infections can occur sporadically or in clusters [5,8]. As mentioned before, the inoculation eschar is the hallmark for this condition and multiple eschars are seen in up to 54% of patients [2]. This may not be evident in patients with dark skin. Neck pain and nuchal stiffness are usually prominent symptoms, and a rash is usually not observed. Aphthous stomatitis is sometimes seen. Considerable clinical overlap occurs between African tick bite fever and the Medititerranean Spotted Fever (*fièvre boutonneuse méditerranéenne*), the latter being caused by *R. conorii *– a strict intracellular bacterium transmitted to humans by the dog tick *Rhipicephalus sanguineus*, mostly in urban settings. While the fatality rate can be up to 4% for Mediterranean Spotted Fever, no fatal case of African tick bite fever has been described.

Although there is no randomised clinical control trial data to guide treatment, therapy with doxycycline 100 mg twice daily for 7–10 days is associated with rapid recovery, within 24–38 hours, in most patients [2]. Occasionally, patients can show a slow response of up to 3–5 days [7]. An interesting finding in our case is that our patient failed to show any improvement after 3 days of therapy with doxycycline. As clinical symptoms were actually more intense on the 3^rd ^day of therapy, therapy was changed to ciprofloxacin, and a rapid response was then observed. Previous reports of clinical failure with doxycycline are rare. Jensenius *et al*. [3] reported that a patient with African tick bite fever complicated by reactive arthritis had no response to 3 days of doxycycline. Similar to our report, the patient's symptoms resolved with the use of ciprofloxacin. Parola *et al*. also described a patient who received doxycycline for 3 weeks and recovered slowly [4]. *In vitro *studies have shown *R. africae *to be susceptibility to both tetracyclines and the fluoroquinolones [9], and *in vitro *resistance to these antibiotics has not yet been demonstrated. The possibility of resistant isolates of Rickettsiae being present in Africa requires further observation and study.

## Conclusion

Spotted fever group rickettsioses are becoming increasingly prevalent amongst travellers to endemic areas in Africa. These conditions are usually not severe and present with unspecific flu-like symptoms. Treatment is relatively simple but requires a high index of suspicion due to the non-specific clinical findings. Important clues for the diagnosis of African tick bite fever are the presence of inoculation eschars (a highly suggestive sign), and the occurrence of the disease in clusters. Although most infected patients promptly recover after therapy when doxycycline is initiated, some patients can be slow- or non-responders. Ciprofloxacin represents an alternative therapy, which was effective in the case of our patient.

## Competing interests

The author(s) declare that they have no competing interests.

## Authors' contributions

All authors provided an equal intellectual contribution to this manuscript. The clinical notes were reviewed by RR. All authors read and approved the final manuscript.

## Consent

Written informed consent was obtained from the patient for publication of this case report and any accompanying images. A copy of the written consent is available for review by the Editor-in-Chief of this journal.
